# Supplementary material for: The impact of authentic leadership on the work engagement of primary and secondary school teachers: The serial mediation role of school climate and teacher efficacy
Source: PLoS One. 2025 May 23;20(5):e0320839. doi: 10.1371/journal.pone.0320839 (PMC12101708; doi:10.1371/journal.pone.0320839)
Supplement: Attachment — Submitted filename: Response to Reviewers.pdf [file pone.0320839.s004.pdf]

We extend our deepest gratitude for the insightful and valuable feedback you have provided. Your expertise and suggestions have been instrumental in enhancing the quality of our manuscript. We have carefully reviewed each of your comments and have made the necessary corrections in accordance with your guidance. Below, we present our point-by-point responses to the comments from each esteemed reviewer:

Reviewer 1

1.Reviewer's comment: "The research significance of this paper needs to be analyzed in depth, especially in the abstract, where it should be clearly stated rather than using vague phrases like "practical implications", "crucial role". For example, in what specific ways can the work engagement of primary and secondary school teachers be improved?"

Our answer: Thank you for your positive feedback and valuable insights.. We have revised the significance of this paper, with the revised parts highlighted in the abstract on Page 1.

2.**Reviewer's comment:** The discussion needs to be analyzed from a more critical perspective. I noticed that much of Section 5 is simply a description of the previous hypotheses, without presenting many novel or critical viewpoints.

Our answer: Thank you for your positive feedback and valuable insights.. We have added critical viewpoints in Section 5. The additional content can be found on Pages 12-14.

3.**Reviewer's comment: "**Another typical example is the statement in the discussion: "schools should invest resources to improve the physical environment of the campus...". This is a very general summary. I believe that schools have limited resources, and these resources may be in competition with one another. For instance, if some funds are allocated to improving the physical environment, then teachers' salaries may be correspondingly reduced. How could this situation be evaluated? Of course, I agree that improving the physical environment is important, but the premise is that the project should have clear goals and a balance with other indicators. The author needs to explain this aspect. In other words, the author not only needs to state the benefits of improving the physical environment but also needs to acknowledge the potential risks involved."

Our answer: Thank you for your positive feedback and valuable insights.. We have added the balance between the physical environment and the intangible aspects that contribute to teacher well-being and job satisfaction. The added content can be found in red on Page 15.

4. **Reviewer's comment:** "I noticed that the author used many uncertain terms to describe the research findings, such as "One possible reason is that authentic leadership ignites teachers' intrinsic motivation, leading them to develop a deeper love for their profession." However, this is not a characteristic of a high-quality paper. The author needs to use clear language to present the research findings, rather than using vague statements. The author should clearly explain how

authentic leadership influences teachers' motivation, which requires a solid literature review. Additionally, I noticed that the author frequently uses "should" to describe conclusions, but if we consider the practice scenario, 'could' would be more appropriate. If 'should' is used, there needs to be sufficient arguments to prove that it is reasonable.

Our answer: Thank you for your positive feedback and valuable insights. We have modified the uncertain terms to describe the research findings and we have replaced "should" with "could " on Pages 15-16.

5. **Reviewer's comment:** Authentic leadership is the focus of this paper's discussion, but after reading it, I believe it leans more towards "political discourse", as the author's definition is quite vague. The author not only needs to discuss the dimensions used to measure authentic leadership but also needs to clarify the reasons for the emergence of authentic leadership. In other words, the author should explain the relationship between authentic leadership and the social system, how the social system fosters authentic leadership, and how authentic leadership drives the social system.

Our answer: Thank you for your positive feedback and valuable insights.. We have replaced "ecological systems theory" with "ecological systems theory" and discussed how authentic leadership affects teachers' work engagement within the framework of ecological systems theory. Please refer to Pages 12-14.

6. **Reviewer's comment:** "The author states, "The social-ecological systems theory posits that individual behaviors are influenced by multiple interconnected levels of their environment, ranging from the immediate setting (microsystem) to the broader societal context (macrosystem)". I must seriously point out that this is not the main viewpoint of the social-ecological systems theory; this concept is more aligned with sociology. I recommend the author study the papers by McCay (2002) and Giddens (1984), which indicate that while individuals are influenced by the structures in which they are embedded, they can also influence and change these structures. I also need to correct an obvious mistake made by the author: the social-ecological systems theory is an academic term in natural resource management, proposed by Elinor Ostrom, primarily discussing the coevolution of social systems and natural systems, with a focus on polycentric governance. Clearly, the use of "the social-ecological systems" in this paper is an inappropriate concept. I suggest that the author reorganize the content of the literature review and maintain a rigorous attitude towards specialized terminology. I have noticed that the author's analysis primarily states that principals need to possess authentic leadership. However, this skill is something that every leader, including grassroots leaders, should have."

Our answer: Thank you for your positive feedback and valuable insights.. We have revised "Social-ecological systems theory" to "The social-ecological systems theory (Bronfenbrenner)" and made several modifications throughout the text. Please see pages 3 and 4 for these changes. Additionally, we have amended the term "principals" to "leaders" on Pages 15-16.

7. **Reviewer's comment: "**In the empirical section, I suggest the author include a "Robustness test" section to verify whether the research results remain stable and reliable under different conditions. This actually addresses my second comment, where the author needs to ensure that the findings are valid under various conditions and can be applied to regions beyond Shandong. The robustness test should primarily include "Sobel verification" and retesting."

Our answer: Thank you for your positive feedback and valuable insights. We have added the section of "Robustness test" on Pages 11-12. In conducting our research, we've come to understand that due to constraints in time and resources, it's not feasible for us to conduct the research again or to introduce new variables. Moreover, the diversity of the groups we are dealing with makes further research quite complex. As a result, we've adopted a pragmatic approach by selecting a key dimension from the authenticity leadership theory to perform stability checks. We believe this method will provide reliable insights under the current circumstances.

Reviewer 2

Reviewer's comment: "Congratulations on the research, I truly believe the study is of very high impact. though i also advise you reassess and write the study to gain its full impact. You have high compiled evident to research but have not imputed it in effective manner, the implication of you study will bring forth changes in viewing teachers and their training to the current educational system. Wishing you the best. "

Our answer: Thank you for your positive feedback and valuable insights. We appreciate your suggestion to reassess and enhance our study's presentation to maximize its impact. We have worked on refining our approach to ensure that our findings are communicated effectively and contribute meaningfully to the current educational discourse.

Reviewer 3

Reviewer's comment: "Very good study having wider implications in policy making. Authentic leadership is the key to ensure quality education in schools. Intrinsic motivation motivated through authentic leadership definitely matters in teacher's work engagement. Appreciate the interest taken in performing this study."

Our answer: Thank you for your positive feedback and valuable insights. We appreciate the reviewer's accepting it in this current form and having no further concerns.
