# Supplementary material for: The impact of authentic leadership on the work engagement of primary and secondary school teachers: The serial mediation role of school climate and teacher efficacy
Source: PLoS One. 2025 May 23;20(5):e0320839. doi: 10.1371/journal.pone.0320839 (PMC12101708; doi:10.1371/journal.pone.0320839)
Supplement: Attachment — Submitted filename: Response_to_Reviewers_auresp_2.pdf [file pone.0320839.s005.pdf]

We extend our deepest gratitude for the insightful and valuable feedback you have provided. Your expertise and suggestions have been instrumental in enhancing the quality of our manuscript. We have carefully reviewed each of your comments and have made the necessary corrections in accordance with your guidance. Below, we present our point-by-point responses to the comments from each esteemed reviewer:

Reviewer 1

Reviewer's comment: The authors need to provide definitions for key terms in the introduction section (or at the point of their first mention).

Our answer: Thank you for your positive feedback and valuable insights.We have provided definitions for key terms in the introduction section. Please take a look at the red text on Pages 2-3 in the introduction section.

Reviewer 2

Reviewer's comment: Hopefully provides better implications for the study in the future.

Our answer: Thank you for your positive feedback and valuable insights. We have added the content on Pages 15-16 in the section of implication.

Reviewer 3

Reviewer's comment: No Response

Our answer: We truly appreciate your positive assessment and the time you've taken to review our work.
