# Supplementary material for: The impact of authentic leadership on the work engagement of primary and secondary school teachers: The serial mediation role of school climate and teacher efficacy
Source: PLoS One. 2025 May 23;20(5):e0320839. doi: 10.1371/journal.pone.0320839 (PMC12101708; doi:10.1371/journal.pone.0320839)
Supplement: Attachment — Submitted filename: PONE-D-24-26731 review.docx [file pone.0320839.s002.docx]

**The impact of authentic leadership on the work engagement of primary and secondary school teachers: The serial mediation role of school climate and teacher efficacy**

Abstract

The authors provide a clear abstract that is structure, the authors could have provided clarity in relation to the assessment tool or the variables aided in providing the objective of the study in method. The result could be concise indicative of significant relations as it comes across as a conclusion of findings.

Introduction

The authors have written a elaborate background to the study along with a review of literature. Given this is a scientific report, they could have combined introduction with the review to narrate flow of information in concise to arriving to an objective that can be followed up with the need for the study. In relation to the hypothesis introduced, the rations are defined though it would be of more clarity in scientific writing for the hypothesis to be defined in the method/ methodology and framing objectives can be of priority in the introduction.

Method

The authors can revise the methodology to provide more structure and clarity. The authors have significantly elaborated on the assessment tools but this could be concise to involve only significant aspects to validate data collected. There is brief description on the study design and sampling though clarity of the processes nit described well. Given its a survey study with stratified sampling – an illustrated process of sampling could be beneficial to clarify the methodology and follow of the study data and analysis.

Results

The description of demographics of the study marks the significance in the survey study. The lack of such emphasis in the results may limit the significance of the study. The authors can describe finding and emphasis on significance before discussion outcomes (outcomes are often observed with literature review and critical evaluation and evidence in discussion.

Discussion

The authors have researched for the discussion, though organization of content along with more referential evaluation of findings based on existing research and associated with the variables of the study. Further following to discuss the limitations and implications of the study.

Note : Authors are advised to review the research writing process to write the paper. The paper has great implications though a more scientific representation may prove valuable in portraying a strong research.
